# Identification of the Beer Component Hordenine as Food-Derived Dopamine D2 Receptor Agonist by Virtual Screening a 3D Compound Database

**DOI:** 10.1038/srep44201

**Published:** 2017-03-10

**Authors:** Thomas Sommer, Harald Hübner, Ahmed El Kerdawy, Peter Gmeiner, Monika Pischetsrieder, Timothy Clark

**Affiliations:** 1 Computer Chemistry Center, Department of Chemistry and Pharmacy, Friedrich-Alexander-Universität Erlangen-Nürnberg, Nägelsbachstr. 25, 91052 Erlangen, Germany; 2 Food Chemistry Unit, Department of Chemistry and Pharmacy, Friedrich-Alexander-Universität Erlangen-Nürnberg, Schuhstr. 19, 91052 Erlangen, Germany; 3 Department of Chemistry and Pharmacy, Emil Fischer Center, Friedrich-Alexander-Universität Erlangen-Nürnberg, Schuhstr. 19, 91052 Erlangen, Germany; 4 Department of Pharmaceutical Chemistry, Faculty of Pharmacy, Cairo University, Kasr-el-Aini Street, Cairo, P. O. Box 11562, Egypt; 5 Molecular Modeling Unit, Faculty of Pharmacy, Cairo University, Kasr-el-Aini Street, Cairo, P. O. Box 11562, Egypt

## Abstract

The dopamine D2 receptor (D2R) is involved in food reward and compulsive food intake. The present study developed a virtual screening (VS) method to identify food components, which may modulate D2R signalling. In contrast to their common applications in drug discovery, VS methods are rarely applied for the discovery of bioactive food compounds. Here, databases were created that exclusively contain substances occurring in food and natural sources (about 13,000 different compounds in total) as the basis for combined pharmacophore searching, hit-list clustering and molecular docking into D2R homology models. From 17 compounds finally tested in radioligand assays to determine their binding affinities, seven were classified as hits (hit rate = 41%). Functional properties of the five most active compounds were further examined in β-arrestin recruitment and cAMP inhibition experiments. D2R-promoted G-protein activation was observed for hordenine, a constituent of barley and beer, with approximately identical ligand efficacy as dopamine (76%) and a K_i_ value of 13 μM. Moreover, hordenine antagonised D2-mediated β-arrestin recruitment indicating functional selectivity. Application of our databases provides new perspectives for the discovery of bioactive food constituents using VS methods. Based on its presence in beer, we suggest that hordenine significantly contributes to mood-elevating effects of beer.

Homeostatic food intake is regulated mainly by the hypothalamus and caudal brainstem by integration of various peripheral and central signals resulting in an ingestive behaviour which counterbalances the energy expenditure^1^. However, it is also well established that certain food stimuli induce hedonic food intake in the state of satiety, leading to an overconsumption of calories and, thus, eventually to obesity. Dopaminergic pathways are heavily involved in hedonic food intake by mediating reward, motivation and reinforcement^2^. Among the five dopamine receptor subtypes, in particular the dopamine D2 receptor (D2R) seems to be involved in food reward and compulsive food intake^2^,^3^. However, the molecular determinants of palatable food inducing non-homeostatic food intake are still not fully understood. Mixtures of carbohydrates and fat most efficiently induce hyperphagia in rats^4^,^5^,^6^, with a carbohydrate/fat ratio of about 45%: 35% having the most pronounced effect^7^. Although being very effective in inducing food intake, fat/carbohydrate mixtures have a lower impact on brain reward areas compared to palatable food items with the same fat/carbohydrate composition^7^,^8^. Thus, bioactive food components with the potential to modulate dopaminergic pathways may be able to change the rewarding properties of food. To date, only little is known about dopaminergic food components. Therefore, the aim of the present study was the identification of novel food-derived D2R ligands.

For the discovery of novel bioactive food components, food extracts or food compounds are either selected hypothesis-driven or even arbitrary and then tested by bioassays addressing the desired physiological effect. Subsequently, active food extracts are subjected to activity-guided fractionation to identify the bioactive components^9^. This approach, however, is rather time-consuming, has a low hit rate and tends to overlook promising novel food bioactives. Furthermore, only a limited number of food components are commercially available for testing or easily accessible. Therefore, *in silico* screening methods would be useful for prioritising the food compounds to be submitted to biological assays.

Virtual screening (VS) refers to the use of computational methods for the knowledge-based identification of compounds that exhibit a desired biological activity^10^. In drug-discovery research, this technique has been developed as a response to stagnating high-throughput screening (HTS) hit rates in combination with rising costs for HTS assays. Nowadays, VS methods are a common complementary technique to HTS to analyse large compound databases in order to prioritise a set of molecules for further experimental testing^11^,^12^,^13^. However, no VS applications in food science have been reported in the literature, possibly because of the lack of specific food-compound databases, since pharmaceutical companies focus on drug-like compounds and natural products. In the present study, we developed a VS protocol consisting of pharmacophore screening, hit-list clustering and molecular docking to search for possible D2R-ligands in a newly assembled database containing 13,000 components from foods and some other natural sources. The most promising compounds were evaluated in biological assays. This approach allowed for the first time the unbiased identification of novel food bioactives by virtual screening of a novel food-compound database.

## Results and Discussion

### Molecular properties of FCDB and PhyDB compared to other VS databases

Our first aim was the generation of an *in silico* 3D food-compound database. Besides this database (FCDB; 12,579 compounds), which we constructed by selecting natural food constituents from the Dictionary of Food Compounds^14^, we also generated a small natural products database (PhyDB; 987 compounds) based on the catalogue from the vendor PhytoLab Vestenbergsgreuth, Germany (available at http://www.phytolab.com/de/phytolab.html). The databases are part of the Supplementary information.

For the characterisation of our newly generated databases, we calculated molecular property distributions for FCDB and PhyDB and for samples containing 10,000 randomly selected compounds from the UNPD database^15^, the ZINC biogenic compounds subset (ZBC) and the ZINC all purchasable subset (ZAP)^16^. Thus, we could compare our databases with established freely available VS databases that contain natural products (UNPD database and ZBC) and drug-like compounds (ZAP). Detailed data are available in the Supplementary information, Fig. S1.

The calculations revealed that the FCDB and PhyDB databases are substantially different to a typical drug-like library (ZAP). While the compounds in drug-like libraries tend to comply with Lipinski’s rule-of-five^17^ resulting in Gaussian-like molecular property distributions with distinct maxima, the molecules in FCDB, PhyDB, and UNPD exhibit very broad molecular-property distributions. Hence, the compounds in these databases tend to have higher structural diversity, which is typical for natural compound libraries^18^. Despite their distinct similarity, differences between FCDB/PhyDB and UNPD still exist, especially in terms of molecular weight. The natural product library ZBC turned out rather to possess drug-like than natural-product-like properties, probably because the authors of ZINC (http://zinc.docking.org/subsets/zbc) took their information about the natural character of a compound from vendor catalogues. The vendors often also include synthetic derivatives of natural products in their catalogues to make them more attractive for medicinal chemists, as also observed by Manallack *et al*.^19^. In summary, it turned out that our generated screening databases represent a new type of screening library, which is much more similar to databases containing natural products than drug-like compounds.

### Virtual screening process

**Figure 1:**
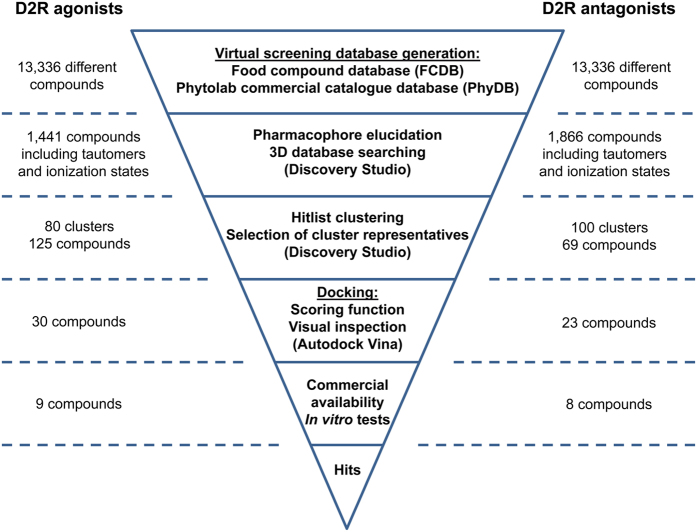
Schematic representation of the VS process from database generation to experimental testing. The numbers on the left and on the right side represent the number of compounds that passed through the respective VS step depicted in the centre. Due to 230 compounds that are present in both FCDB and PhyDB, the absolute number of different compounds is smaller than the sum of the compounds in the two databases.

The next goal was the search for nutritive or natural D2R-ligands in the generated VS databases. D2R is a target of great interest for the pharmaceutical industry, for which agonists have been developed as drugs for treating Parkinson’s disease and antagonists for treating schizophrenia^20^,^21^. Although no X-ray structure of D2R has been published yet, a wide variety of ligands has been synthesised that facilitates the effective use of ligand-based VS methods such as pharmacophore searching^11^. The GPCR Ligand Library^22^ provides a comprehensive collection of known D2R-ligands compiled from the GLIDA database^23^. However, since the majority of D2R-ligands are synthetic in origin, the ligand collections contain primarily drug-like compounds, in contrast to those contained in FCDB and PhyDB. Therefore, the selected VS methods needed to be capable of finding VS hits in FCDB and PhyDB that are structurally different to the input ligands. Therefore, we did not only apply VS methods that rely on molecular similarity but rather used pharmacophore searching in combination with hit-list clustering and molecular docking. The overall VS workflow is summarised in Fig. 1.

### Pharmacophore modelling

By selecting relatively large and structurally diverse training sets for both D2R-agonists and -antagonists, we ensured that the resulting pharmacophore models only retain the most critical features for binding. The training sets are depicted in the Supplementary information, Figs S2 and S3. Training sets containing only large and structurally similar compounds would probably have resulted in very specific pharmacophore models with a large number of features, which would not be able to find any hits in FCDB and PhyDB. Additionally, we did not consider excluded volumes in the pharmacophore models to avoid obtaining small hit lists.

It is a prerequisite for an alignment of the ligands to be a good approximation to their binding mode that the template compound for the alignment provides an orientation close to its bioactive conformer. In addition to molecular docking and subsequent molecular-dynamics refinement of the ligand conformers as techniques for approaching the bioactive conformation, we selected some ligands, namely # **21** and # **27** from the agonist training set, Fig. S2, and # **32** from the antagonist training set, Fig. S3. We used the conformers that are stored in the GPCR Ligand Library directly, because the conformational space in low-energy regions is sparsely occupied for structurally restrained ligands. The bioactive conformer has been shown to be often located near the global minimum^24^ and a low-energy conformer for a restrained ligand can thus be a good approximation. The training set ligand conformers that possess the maximum similarity compared to the template were then determined by ParaAlign (see Methods and Supplementary information). For each ligand, this conformer was extracted and used as input for the HipHop^25^ pharmacophore model generation algorithm. Since we generated up to 25 pharmacophore models per run, the variation of the conformer types and the templates that were used for ParaAlign resulted in a large number of models, which had to be validated subsequently.

**Figure 2:**
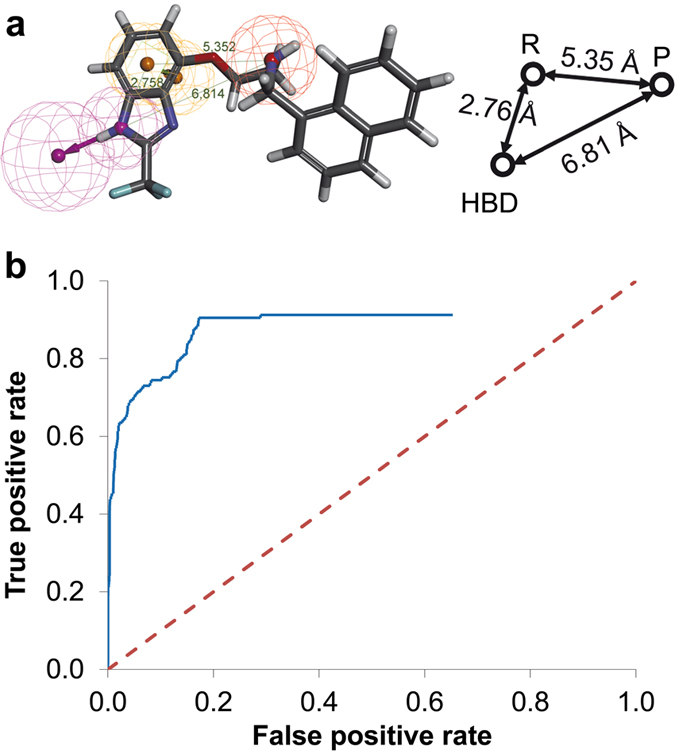
The overall best D2R-agonist pharmacophore and its inter-feature distances (**a**), depicted together with its ROC curve that results from VS of the test database (**b**). The features are hydrogen bond donor (purple, HBD), aromatic ring (orange, R) and positive ionisable (red, P). The dashed red line in the ROC plot indicates a random selection.

The validation process involved large test databases compiled from the GPCR Ligand Library and GPCR Decoy Database^22^ in order to determine the overall best pharmacophore models. We found two very similar pharmacophore models with almost identical excellent VS performance in model validation for the D2R-agonists. Both models contained hydrogen-bond donor, aromatic ring and positive ionisable features. We therefore decided to use only the model that gave a slightly better receiver operating characteristic (ROC) curve (Fig. 2b).

A comparison between this D2R-agonist pharmacophore model (Fig. 2a) and models reported in the literature revealed a high degree of similarity. Both Malo *et al*.^26^ and Chidester *et al*.^27^ reported D2R-agonist pharmacophore models that also contain hydrogen-bond donor, aromatic ring and positive ionisable features with similar relative orientations. The only difference is an additional hydrophobic feature close to the positive ionisable feature present in their models. However, since not all our training set ligands contain such a feature, we conclude that the hydrophobic feature is able to increase binding affinity but that it is not critical for binding.

**Figure 3:**
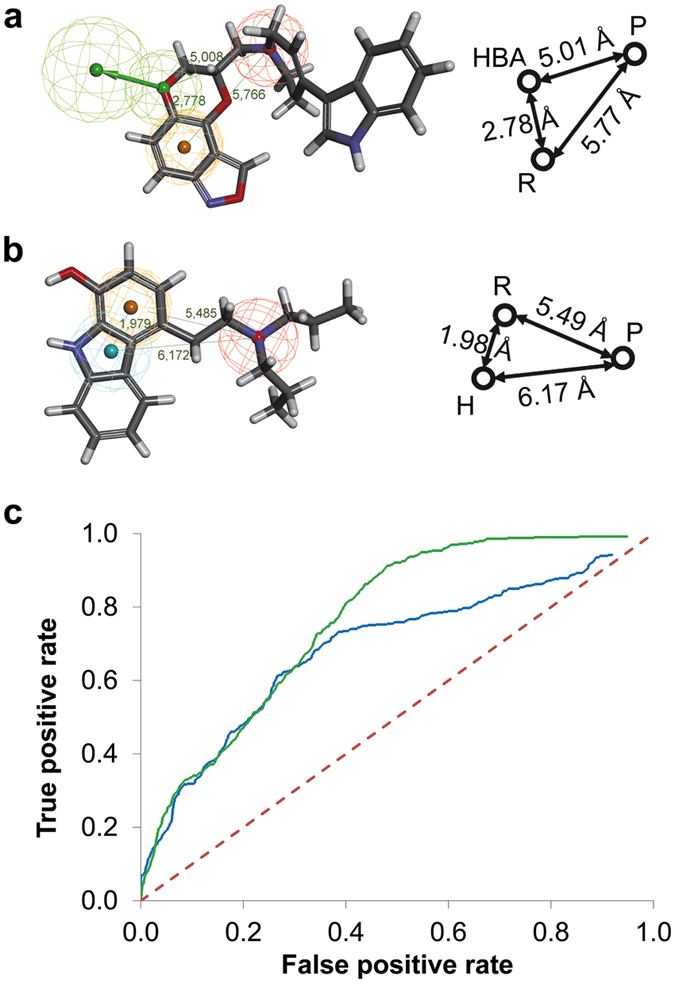
The overall best D2R-antagonist pharmacophore models and their inter-feature distances (**a** and **b**) depicted together with the corresponding ROC curves that result from VS of the test database (**c**). The features are hydrogen bond acceptor (green, HBA), aromatic ring (orange, R), positive ionisable (red, P) and hydrophobic (blue, H). The dashed red line in the ROC plot indicates a random selection. The blue curve corresponds to model a) and the green curve to model b).

We also obtained two pharmacophore models for the D2R-antagonists that exhibited the best VS performance in the validation step (Fig. 3c). In contrast to the agonists, these two models did not show a high degree of similarity concerning shape and features (Fig. 3a,b). While the first D2R-antagonist model contained a hydrogen-bond acceptor, an aromatic ring and a positive ionisable feature, the other model possessed a hydrophobic feature together with the aromatic ring and positive ionisable feature. The relative orientation of the features was also different. However, this observation is not surprising, since our D2R-antagonist training set covered ligands with considerably different shapes (e.g. ligands # **35** and **39,** Fig. S3). Such diverse ligands probably adopt different binding modes and, hence, it is not surprising that we obtained two diverse pharmacophore models.

The models further resemble a ligand classification for pharmacophore model generation reported by Klabunde and Evers^28^. They divided their D2R-antagonist training set into two different classes and built different pharmacophore models from the two subsets. Our first D2R-antagonist pharmacophore model (Fig. 3a) resembles their class I model, whereas our second model (Fig. 3b) shows some similarity to their class II model. As our validation database contains a substantial number of actives from both ligand classes, similar VS performance of the pharmacophore models was not surprising. The validation performance is not as good as for the D2R-agonist models, probably because, on the one hand, either model is recognising class I-like actives or class II-like actives better and, on the other hand, hydrophobic and hydrogen-bond acceptor features are not as selective as a hydrogen-bond donor feature. For this study, we decided to use both models for database searching in FCDB and PhyDB and, subsequently, to combine the two hit lists to avoid missing any type of antagonist.

The 3D database-searching step using the selected D2R-agonist pharmacophore model selected 1,441 compounds from FCDB and PhyDB. In contrast, the two selected D2R-antagonist pharmacophore models gave a combined hit list with 1,866 compounds. Note that we treated tautomeric compounds and different ionisation states as different database entries in this step, which led to a lower number of chemically different hit compounds.

### Hit-list clustering and compound prioritisation

The next goal of our study was to select structurally diverse compounds for experimental testing. Therefore, we performed a clustering analysis of the hit lists in Discovery Studio 3.1 (Accelrys Inc., San Diego, CA) to divide them into groups that contain structurally different classes (Fig. 1). Moreover, the possible corresponding tautomers and ionisation states for a given hit compound could be pooled as they were assigned to the same compound cluster. After variation of the number of generated clusters, we found that values of 80 clusters for the D2R-agonist hits and 100 clusters for the D2R-antagonist hits represent good values for characterising the hit-list compounds.

Selecting compounds from different clusters ensured that we chose structurally diverse molecules for the following molecular-docking step. The choice was further based on the fit value to the pharmacophore models and on visual inspection. For both D2R-agonist and -antagonist screening, we derived a cut-off limit for the fit value from the pharmacophore validation step, in which databases consisting of known actives and putative inactives were mapped to the models. From the actives mapped to the D2R-agonist model, all but one gave a fit value higher than 2.2. Hence, we used this value as a cut-off limit in compound prioritisation. By contrast, most of the actives mapped to the D2R-antagonist models showed fit values of 1.9 and 2.3 for model A and model B, respectively. We retained pharmacophore searching hits with larger fit values than these cut-off limits for at least one of the models.

Hit-list clustering and compound prioritisation reduced the number of VS hits to 125 and 69 different compounds for the D2R-agonist and -antagonist screening, respectively. Thus, we selected less compounds from the D2R-antagonist screening for the molecular docking step, although the pharmacophore hit list and the number of clusters was larger than for the agonists. However, many compounds in the antagonist hit-list did not pass the fit-value cut-off. Additionally, visual inspection of the hit-list molecules revealed that large and bulky compounds tended to have very high fit values. These molecules were rejected because they are unlikely to fit into the binding pocket.

### Molecular docking and final compound selection

The combination of pharmacophore searching with molecular docking enabled an initial check of our database molecules for the required molecular features and their relative orientations. Then we examined more thoroughly, whether the candidates could fit well into the binding pocket and establish important protein-ligand interactions. A D2R inactive-state homology model used successfully in a previous docking study^29^ served as docking target. The compounds that passed the clustering and prioritisation step were docked together with their corresponding tautomers and ionisation states using AutoDockVina^30^ with high exhaustiveness and maximum conformer values for detailed examination. For each compound, we carefully inspected both the value of the scoring function and the resulting receptor-ligand complexes. An interaction between a positively charged moiety and the carboxylate group of Asp114^3.32^ was mandatory for a compound to be regarded as hit in the docking step.

**Figure 4:**
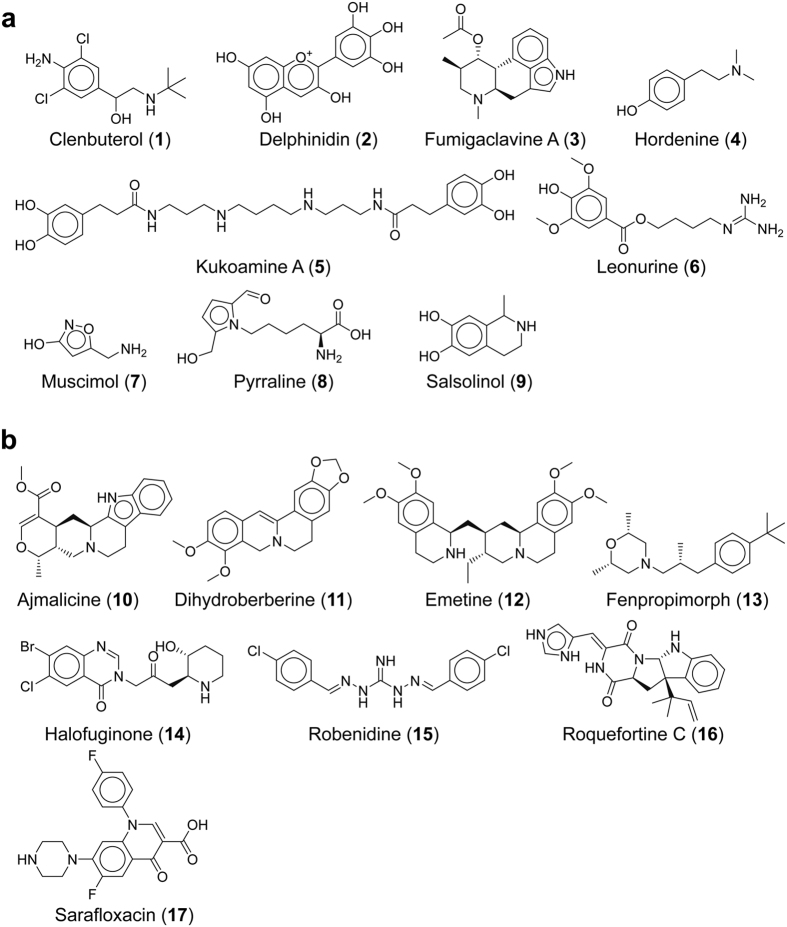
Structures of the experimentally tested D2R-agonist (**a**) and -antagonist (**b**) VS hits. The 17 compounds can be classified into natural food constituents (delphinidin, hordenine, kukoamine A, pyrraline, salsolinol, roquefortine C), further compounds from natural sources (fumigaclavine A, leonurine, muscimol, ajmalicine, dihydroberberine, emetine), and food contaminants (clenbuterol, fenpropimorph, halofuginone, robenidine, sarafloxacin).

Eventually, we obtained 53 hit compounds and searched for corresponding possible activity data concerning dopamine receptors in the literature. We excluded hits with known bioactivity at D2R and checked the commercial availability of the remaining compounds. Finally, 17 compounds of different origin (Fig. 4a,b, Table 1) were purchased and prepared for experimental testing.

We also examined, whether a 2D molecular similarity search would have been sufficient to extract these molecules from the VS databases. Therefore, we calculated Extended Connectivity Fingerprint 4 (ECFP4)-based Tanimoto coefficients (T_c_) for the nine D2R-agonist screening hits and the eight D2R-antagonist screening hits compared to the respective training set ligands. For the D2R-agonist hits, the T_c_ values varied from 0.094 (pyrraline) to 0.324 (salsolinol), while we obtained T_c_ values between 0.110 (fenpropimorph) and 0.345 (ajmalicine) for the D2R-antagonist hits. When comparing all our screening database compounds to the training set ligands, the top 100 hits using the D2R-agonist training set as a reference gave T_c_ values of at least 0.234, while the T_c_ values of the top 100 hits using the D2R-antagonist training set as a reference were at least 0.243. Thus, when applying the 2D molecular similarity search, only three of our selected compounds, ajmalicine (T_c_ = 0.345), salsolinol (T_c_ = 0.324) and fumigaclavine A (T_c_ = 0.288) would have been present among the top-ranked compounds. For details, please refer to the Supplementary information, Table S1.

### Experimental evaluation

To confirm the VS predictions, we conducted radioligand binding assays to estimate the binding affinity of our hit compounds at D2R. As we performed VS to identify both nutritive D2R-agonists and -antagonists, we were interested in the ability of the VS hits to activate the receptor or to antagonise the effects of dopamine. Hence, functional assays were carried out to evaluate both G protein- and β-arrestin-mediated signalling pathways.

### Ligand binding experiments

The affinity profiles of our VS hits towards the human dopamine D_2L_ receptor isoform were compared with those of the reference compound dopamine by radioligand binding experiments (Table 1).

The binding data revealed K_i_ values ≤ 50 μM for five of nine tested D2R-agonist VS hits, representing a hit rate of 56%. Two out of eight tested D2R-antagonist VS hits gave K_i_ values ≤ 50 μM corresponding to a 25% hit rate. All observed binding affinities were in the micromolar range, which is typical for active compounds found by VS^31^. Fumigaclavine A, an alkaloid with an ergoline-like structure produced by different *Aspergillus* species^32^, showed the highest binding affinity with a K_i_ value that was only seven times higher than for the endogenous ligand dopamine. Furthermore, the coccidiostat and possible food contaminant robenidine^33^, as well as salsolinol and hordenine bind in the low micromolar range. Salsolinol, for which controversial activity profiles for D2 receptors have been reported^34^,^35^,^36^, is a constituent of cocoa and chocolate^37^, whereas hordenine is present in barley^38^ and beer^39^. The anthocyanidin delphinidin, which has a considerably different molecular scaffold to the remaining VS hits, gave a K_i_ value of 26 μM. Most active compounds contain an aliphatic amine as a positively charged moiety at physiological pH to enable a salt bridge to Asp114^3.32^. Only robenidine, which contains a guanidinium group, and delphinidin, which features cationic oxygen, are different.

### Functional experiments

Finally, we examined the ability of our VS workflow not only to identify compounds that bind to D2R, but also to predict the compound efficacy. Hence, we performed functional experiments for the five compounds that showed the best binding affinities, namely fumigaclavine A, robenidine, salsolinol, hordenine, and delphinidin. As it has been shown that ligands can induce G protein-mediated signalling and β-arrestin recruitment independently^40^,^41^, the individual pathways were investigated separately (Table 2).

**Figure 5:**
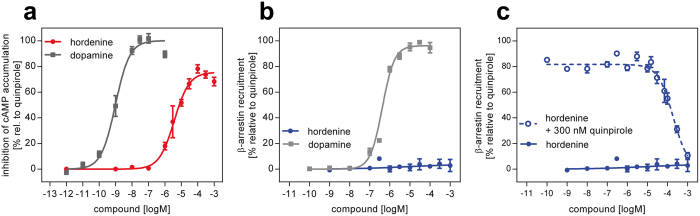
Receptor activation properties of hordenine. In comparison to dopamine, hordenine shows agonist properties in the inhibition of forskolin-stimulated cAMP accumulation (**a**). While no β-arrestin-2 recruitment was determined for hordenine (**b**), the test substance completely antagonised quinpirole-stimulated recruitment (**c**).

Among the five investigated VS hits, three compounds showed significant intrinsic activity in the cAMP accumulation assay. The EC_50_ values of fumigaclavine A and hordenine are both in the extended range of the K_i_ values determined in the radioligand binding experiments. Conversely, probably because of a very flat dose-response curve, the EC_50_ value of salsolinol is considerably lower than its K_i_ value. For hordenine, we could observe almost full activation (E_max_ = 76%) compared to the reference compound quinpirole and to dopamine (Fig. 5a). This is particularly interesting, because hordenine did not show significant β-arrestin recruitment (Fig. 5b). In fact, titration curves using a constant concentration of 300 nM of the D2R-agonist quinpirole with hordenine confirm its antagonistic activity (Fig. 5c). Fumigaclavine A and salsolinol also did not show β-arrestin recruitment, but the maximum response of these compounds in the cAMP accumulation assay was well below 50%, categorising them as weak partial agonists.

**Figure 6:**
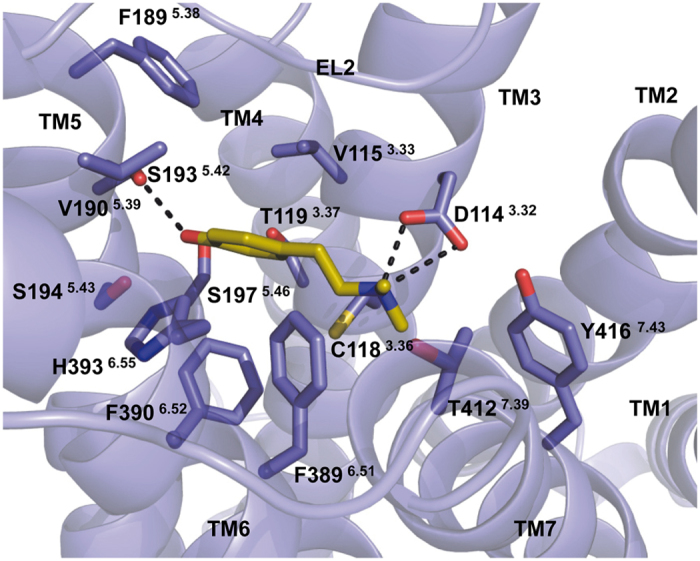
Conformation of hordenine and its receptor-ligand interactions obtained after docking and energy minimisation. We used an active-state homology model of D2R and performed MD simulations with the endogenous ligand dopamine^42^. Dopamine was removed from the model, hordenine was docked into the binding pocket and the resulting receptor-ligand complex was subjected to energy minimisation in a water box. Whereas dopamine is able to form two hydrogen bonds with both Ser193^5.42^ and Ser197^5.46^ in the D2^Up^R model^42^, our VS hit hordenine forms only a single hydrogen bond to Ser197^5.46^ due to the lack of a second hydroxyl group.

Although the G protein-biased D2R-agonist hordenine shares structural similarities to the balanced agonist dopamine, receptor–ligand interactions obtained after docking and energy minimisation in presence of a D2R homology model appear to be different. Hordenine lacks a *meta*-hydroxyl group compared to dopamine, rendering it unable to form hydrogen bonds to both residues Ser193^5.42^ and Ser197^5.46^ as dopamine does^42^ (Fig. 6). The receptor–ligand complex that results from docking and energy minimisation cannot explain whether double methylation of the nitrogen atom enables hordenine to form additional hydrophobic interactions compared to dopamine.

Taken together, we could observe partial or almost full agonist activity in the cAMP inhibition assays for three of four tested D2R-agonist VS hits. For the D2R-antagonist VS hit robenidine, we could not observe any activation in either assay. These results confirm the pharmacophore models and VS workflow.

Thus, the VS workflow proved to be able to identify hordenine as almost full D2R-agonist in G_i/o_ activation and simultaneous antagonism in β-arrestin recruitment, indicating that this substance acts as a G protein-biased agonist at D2R. Additionally, two VS hits with partial agonism in G_i/o_ activation, fumigaclavine A and salsolinol, were identified. The D2R-antagonist robenidine did not show any activation in either assay. The VS method based on the newly generated VS food database can now be applied to other targets for unbiased identification of novel bioactive compounds in food.

Hordenine is a natural constituent of barley and, in particular, beer, a food that is often linked to alcohol abuse. In the present study, hordenine was able to bind to D2R with a K_i_ value in the low micromolar range. The activation profile of hordenine showed significant bias for G protein-promoted activation over β-arrestin recruitment compared to functionally balanced D2R-agonists like the endogenous ligand dopamine. Given that β-arrestin recruitment leads to desensitisation and receptor internalisation, hordenine-promoted D2R activation may be stronger and more sustainable than activation with the balanced neurotransmitter dopamine. Further studies are necessary to clarify the possible influence of hordenine on the dopaminergic system and food reward.

## Methods

### Database generation

From the 40,000 entries (natural food constituents, food contaminants, food additives, nutraceuticals) in the Dictionary of Food Compounds^14^ only those molecules were selected for FCDB that were reported in the literature to occur in food. We excluded compounds that were heavier than 750 Da or possessed more than two sugar moieties, because they are unlikely to be resorbed in the gastrointestinal tract. The Supplementary information includes further details on the database generation procedure.

### Molecular property calculations

For comparison of FCDB and PhyDB with other established database types, we selected the UNPD database^15^, the ZINC biogenic compounds (ZBC), and the ZINC all purchasable (ZAP) subset from the ZINC database^16^. From these three databases, we extracted a sample of 10,000 random compounds each. By contrast, FCDB and PhyDB were used in their entirety for the calculation of molecular properties. The molecular properties (molecular weight, AlogP, numbers of rotatable bonds, hydrogen bond donors and acceptors) were calculated in Discovery Studio 3.1.

### Pharmacophore modelling

For pharmacophore model generation, we selected structurally diverse training sets containing 11 D2R-agonists and 12 D2R-antagonists collected from the GPCR Ligand Library^22^. To obtain low-energy conformers for the training set ligands, we generated conformational sets with up to 255 conformers using the Catalyst BEST conformer generation algorithm (once including and once without a conformer minimisation step using the CHARMm force field implemented in Discovery Studio 3.1.) The generated ligand conformers were aligned to a template compound, which is assumedly present in its bioactive conformation.

To obtain template conformers for the rigid-body alignment algorithm ParaAlign (detailed description of ParaAlign is given in the Supplementary information), we submitted each training set ligand to a molecular docking step into an in-house D2R homology model using AutoDock Vina^30^ followed by molecular dynamics refinement of the ligand conformers in the receptor-ligand complex using AMBER10 programme package (University of California). The refined docking conformation of each training set ligand was subsequently used as template for ParaAlign. Because our training sets also contained structurally restrained ligands, we additionally used the ligand conformers for these compounds that are stored in the GPCR Ligand Library directly as templates for ParaAlign. Two agonists (# **21** and **27**, Fig. S2) and one antagonist (# **32**, Fig. S3) underwent this procedure. Using the ParaAlign algorithm, we could then determine and extract the respective training set conformer for each alignment that possessed the highest similarity to the template conformer. Subsequently, pharmacophore models were generated for those training set conformers using the Common Feature Pharmacophore (HipHop) algorithm in Discovery Studio 3.1. The applied parameters are given in the Supplementary information.

In a validation step consisting of a database search with test databases that contain a large number (136 agonists, 493 antagonists, and 39 decoys for each) of chemically diverse ligands and suitable corresponding decoys compiled from the GPCR Ligand Library and GPCR Decoy Database, we evaluated all generated pharmacophore models. The overall best D2R-agonist and -antagonist pharmacophore models were extracted for the pharmacophore screening in FCDB and PhyDB using the best flexible 3D database search in Discovery Studio 3.1.

### Hit-list preparation and molecular docking

The hit lists resulting from the pharmacophore searches were merged and duplicate structures were removed. In the following, we applied the ligand-clustering tool in Discovery Studio 3.1 using MDL public key fingerprints as clustering properties to divide the hit lists into groups of structurally similar molecules. Thus, a total number of 80 clusters for the D2R-agonist and 100 clusters for the D2R-antagonist screening hits were generated.

To obtain structurally diverse screening hits for the subsequent molecular docking step, we picked out single cluster representatives based on the pharmacophore-model fit values and on visual inspection. For chiral compounds, we checked if the configuration generated by CORINA corresponded to the natural stereoisomer. If this was not the case, we produced the natural stereoisomer in ChemDraw 12.0, converted it to a SMILES string and to a 3D conformer by CORINA and submitted it to the ligand-preparation steps previously applied to all database compounds. Ligand conformers were generated and mapped to the pharmacophore models to check, if a match was still present.

Subsequently, the selected pharmacophore hits were docked into an in-house D2R-homology model previously used in molecular docking studies^29^ using AutoDock Vina^30^. We applied a search space of 26 × 24 × 24 Å and an exhaustiveness value of 32 and obtained up to 20 conformations of each ligand. For the final selection of screening hits to be tested *in vitro*, the resulting protein-ligand complexes were subjected to an accurate visual inspection. We retained only poses, where the most important receptor-ligand interactions such as the conserved salt bridge between Asp114^3.32^ and a positively charged moiety were present. After checking the final hit lists for commercial availability, 17 compounds were purchased. The VS hit hordenine was further docked into a D2R-homology model used in molecular-dynamics studies with the endogenous ligand dopamine^42^. The docking procedure is described in detail in the Supporting information.

### VS hit compounds

Ajmalicine, delphinidine chloride, dihydroberberine, emetine dihydrochloride, hordenine, leonurine, and muscimol were purchased from PhytoLab GmbH (Vestenbergsgreuth, Germany). Clenbuterol hydrochloride, fenpropimorph, halofuginone hydrobromide, robenidine hydrochloride, and sarafloxacin hydrochloride hydrate were obtained from Sigma Aldrich (Taufkirchen, Germany). Fumigaclavine A was purchased from AdipoGen AG (Liestal, Switzerland), and kukoamine A from AChemTek, Inc. (Worcester, MA, USA). Pyrraline was provided by PolyPeptide Group (Strasbourg, France). Roquefortine C was obtained from Cfm Oskar Tropitzsch GmbH (Marktredwitz, Germany) and salsolinol hydrochloride from ABCAM biochemical (Cambridge, U.K.).

### Radioligand binding assays

Receptor binding studies were carried out as described previously^43^,^44^. In brief, preparations of membranes from CHO cells that stably express the human D_2L_ receptor were used together with [^3^H]spiperone (specific activity 73 Ci/mmol, Biotrend, Cologne, Germany) at a final concentration of 0.15 nM. The assays were carried out at a protein concentration of 6 μg/assay tube, showing K_D_ values of 0.052–0.10 nM and corresponding B_max_ values of 950–1500 fmol/mg.

### cAMP Inhibition assay

HEK293T cells were transiently co-transfected with pcDNA3L-His-CAMYEL^45^ (purchased from ATCC, Manassas, VA via LGC Standards, Wesel, Germany) and D_2s_ receptor, respectively, and the assay was performed according to literature^46^. Twenty-four hours after transfection cells were split into white half-area 96-well plates at 2 × 10^5^ cells/well and grown overnight in a phenol-red free medium supplemented with serum (Invitrogen, Darmstadt, Germany). On the day of measurement, the medium was removed and replaced by phosphate buffered saline (PBS). The cells were serum-starved for 1 h before treatment. The assay was started by adding 10 μL of coelenterazine-h (Promega, Mannheim, Germany) to each well to yield a final concentration of 5 μM. After 5 min of incubation, test compounds were added in PBS containing forskolin at a final concentration of 10 μM. After an incubation time of 15 min, BRET measurement was performed with a CLARIOstar plate reader (BMG LabTech, Ortenberg, Germany). Emission signals from Renilla luciferase and YFP were measured simultaneously using a BRET1 filter set (475–30 nm/535–30 nm). BRET ratios (emission at 535 ± 30 nm/emission at 475 ± 30 nm) were calculated and dose-response curves were fitted by nonlinear regression analysis using the algorithm of PRISM 6.0. Curves were normalised to basal BRET ratio obtained from dPBS (0%) and the maximum effect of the reference ligand quinpirole (100%).

### β-Arrestin recruitment assay

The measurement of β-arrestin-2 recruitment stimulated by D2R activation was performed using the PathHunter® assay purchased from DiscoveRx (Birmingham, U.K.) according to the manufacturer’s protocol as described previously^47^. To determine the antagonist properties, different concentrations of the test compounds (10^−10^–10^−3^ M) were pre-incubated for 30 min before adding 300 nM (final concentration) quinpirole to start the standard incubation period.

### Data analysis

The resulting competition curves of the receptor binding experiments were analysed by nonlinear regression using the algorithms in PRISM 6.0 (GraphPad Software, San Diego, CA). The data were initially fit using a sigmoid model to provide an IC_50_ value, representing the concentration corresponding to 50% of maximal inhibition. IC_50_ values were transformed to K_i_ values according to the equation of Cheng and Prusoff^48^.

Data from cAMP measurements were analysed by normalising the BRET ratios with 0% for the unstimulated receptor and 100% for the full effect of the reference ligand quinpirole. Dose-response curves were calculated by nonlinear regression using the algorithms of PRISM 6.0.

The amount of recruitment of β-arrestin was derived from the agonist-induced increase of chemiluminescence, which was expressed in counts per second (cps). Dose-response curves were normalised to basal cps stimulated by buffer (=0%) and the effect of the maximum effect of the reference compound quinpirole (=100%).

From each curve, a set of mean values was derived and pooled to result in an average curve showing the EC_50_ and E_max_ value, respectively.

## Additional Information

**How to cite this article:** Sommer, T. *et al*. Identification of the Beer Component Hordenine as Food-Derived Dopamine D2 Receptor Agonist by Virtual Screening a 3D Compound Database. *Sci. Rep.* **7**, 44201; doi: 10.1038/srep44201 (2017).

**Publisher's note:** Springer Nature remains neutral with regard to jurisdictional claims in published maps and institutional affiliations.

## Supplementary Material

Supplementary Information

Supplementary Dataset 1

Supplementary Dataset 2

## Figures and Tables

**Table 1:** Compound sources, pharmacophore searching fit values and radioligand binding data for the tested VS hits compared to the reference compound dopamine, employing D_2L_ receptors expressed in CHO cells.

Compound	Compound origin	Ref.	Pharmacophore model fit value^a^		K_i_ ± SEM [nM]^b^ [^3^H]spiperone D_2L_
Dopamine					160 ± 31
**D2R-agonist hits**					
Clenbuterol	Food contaminant, illegal use in calf fattening	49	2.488		50,000 ± 13,000
Delphinidin	Anthocyanidin, in various plants and fruits	50	2.396		26,000 ± 6,800
Fumigaclavine A	Produced by *Aspergillus*	32	2.901		1,100 ± 160
Hordenine	Barley and beer	38,39	2.300		13,000 ± 2,700
Kukoamine A	Potato tubers	51	2.967		>100,000
Leonurine	*Leonotis* species	52	2.833		>100,000
Muscimol	*Amanita muscaria*	53	2.218		>100,000
Pyrraline	Maillard product from glucose and lysine	54	2.853		>100,000
Salsolinol	Cocoa and chocolate	37	2.397		7,300 ± 1,900
**D2R-antagonist hits**			Model A	Model B	
Ajmalicine	*Rauvolvia serpentina*	55	2.287	2.672	>100,000
Dihydroberberine	*Berberis* species	56	2.360	2.252	75,000 ± 27,000^c^
Emetine	*Carapichea ipecacuanha*	57	2.848	2.652	37,000 ± 5,700^c^
Fenpropimorph	Food contaminant, fungicide	58	1.403	2.687	>100,000
Halofuginone	Food contaminant, coccidiostat	59	2.818	2.774	>50,000
Robenidine	Food contaminant, coccidiostat	33	2.163	2.553	5,400 ± 1,600
Roquefortine C	Blue cheese	60	1.087	2.362	>100,000
Sarafloxacin	Food contaminant, antibiotic	61	1.279	2.852	>100,000

^a^The fit value indicates how well a compound matches the pharmacophore model. For our models, which each contain three features, the maximum possible fit value for a compound is 3.

^b^K_i_ values ± standard error of mean (SEM) in nM derived from at least four individual experiments each performed in triplicate.

^c^K_i_ values ± standard deviation (SD) in nM derived from two individual experiments each performed in triplicate.

**Table 2:** Intrinsic activities and potencies of the five most active VS hits determined at the dopamine receptor subtype D_2S_ by measuring the inhibition of forskolin-stimulated cAMP accumulation and recruitment of β-arrestin-2 after stimulation of the D_2S_ receptor expressed in HEK cells^a^.

Compound	Inhibition of forskolin-stimulated cAMP accumulation		β-Arrestin recruitment^b^	
	EC_50_ (nM)	E_max_ (%)^c^	EC_50_ (nM)	E_max_ (%)^d^
Delphinidin	N/A	20% at 100 μM	N/A	<7
Fumigaclavine A	250	27	N/A	<7
Hordenine	3700	76	N/A	<7
Salsolinol	630	21	N/A	<7
Robenidine	N/A	<5	N/A	<7
Dopamine	0.97	100	420	96

^a^EC_50_ and E_max_ values were derived from mean curves based on 3–7 individual dose-response curves.

^b^D_2S_-mediated recruitment of β-arrestin-2 determined with the PathHunter assay.

^c^Maximum effect of forskolin-stimulated cAMP inhibition relative to the effect of quinpirole.

^d^Maximum effect of D_2S_-mediated β-arrestin recruitment relative to the maximum effect of quinpirole. N/A: EC_50_ values could not be analysed because of low corresponding E_max_ values.
