# Supplementary material for: Identification of the Beer Component Hordenine as Food-Derived Dopamine D2 Receptor Agonist by Virtual Screening a 3D Compound Database
Source: Sci Rep. 2017 Mar 10;7:44201. doi: 10.1038/srep44201 (PMC5345022; doi:10.1038/srep44201)
Supplement: Supplementary Information [file srep44201-s1.pdf]

Supplementary information

Identification of the Beer Component Hordenine as Food-Derived Dopamine D2 Receptor Agonist by Virtual Screening a 3D Compound Database

Thomas Sommer[1, 2], Harald Hübner[3], Ahmed El Kerdawy[4,5], Peter Gmeiner[3], Monika Pischetsrieder[2] & Timothy Clark[1]*

[1] Computer Chemistry Center, Department of Chemistry and Pharmacy, Friedrich-Alexander-Universität Erlangen-Nürnberg, Nägelsbachstr. 25, 91052 Erlangen, Germany

[2] Food Chemistry Unit, Department of Chemistry and Pharmacy, Friedrich-Alexander-Universität Erlangen-Nürnberg, Schuhstr. 19, 91052 Erlangen, Germany

[3] Department of Chemistry and Pharmacy, Emil Fischer Center, Friedrich-Alexander-Universität Erlangen-Nürnberg, Schuhstr. 19, 91052 Erlangen, Germany

[4] Department of Pharmaceutical Chemistry, Faculty of Pharmacy, Cairo University, Kasr-el-Aini Street, Cairo, P. O. Box 11562, Egypt

[5] Molecular Modeling Unit, Faculty of Pharmacy, Cairo University, Kasr-el-Aini Street, Cairo, P. O. Box 11562, Egypt

*Correspondence to M.P., e-mail: monika.pischetsrieder@fau.de;

Contents:

A Method details

B Table S1: Molecular similarities for the tested virtual screening hits compared to the respective training set ligands expressed as Tanimoto coefficient T_c, calculated using Extended Connectivity Fingerprints 4 (ECFP4)

C Figures

Figure S1: The molecular property distributions of the newly generated screening databases depicted together with those of other types of freely available virtual screening databases

Figure S2: Training set ligands for D2R-agonist pharmacophore model generation

Figure S3: Training set ligands for D2R-antagonist pharmacophore model generation

D Databases

Method details

Expanding the methods section in the main text, we add further details of the applied virtual screening method.

Database generation

The InChI strings given by the Dictionary of Food Compounds[1] were first curated before 3D structure generation. Erroneous E/Z-configurations and amide tautomers, which are entirely represented as iminol structures, were corrected. The InChI strings do not contain any stereochemical information. Initially, we therefore did not search for the natural stereoisomer, but generated a random stereoisomer to be stored in the database instead. When retrieving chiral compounds as hits in the VS process, the CAS number (provided by the Dictionary of Food Compounds) allows searching for the natural stereoisomer, which can then be re-submitted to the VS workflow. Because the structure generator CORINA (available at http://www.molecular-networks.com) cannot directly convert InChI to 3D structures, we first converted the InChI strings into SMILES strings by ChemDraw 12.0 and used those subsequently as input for CORINA. The final version of FCDB contains 12,579 different compounds, each one with a numerical index and compound name. The following compound-preparation steps involved tautomer generation, protonation at physiological pH and energy minimisation using the CHARMm force field. Afterwards, we calculated low-energy conformational sets using the BEST conformer generation algorithm with up to 150 conformers for every molecule and stored them in a 3D conformer database in Discovery Studio 3.1.

The considerably smaller size of the PhytoLab compound catalogue allowed us to generate the natural stereoisomers in ChemDraw 12.0 first for our natural compound VS database PhyDB. Hence, we used isomeric SMILES strings directly as input for CORINA. The compound-preparation steps were identical to those used for FCDB. The final version of PhyDB contains 987 different compounds.

ParaAlign algorithm

ParaAlign is a rigid-body alignment algorithm that uses electron density (ρ)[2] and molecular electrostatic field (first derivative of the molecular electrostatic potential)[3,4] calculated on a grid around the ligands to be aligned using semi-empirical molecular orbital theory. The alignment algorithm depends on maximising the similarity between the template molecule conformer and the other dataset ligand conformers. The similarity between two molecules' properties on the grid is calculated using Hodgkin's similarity index[5], and the Simplex algorithm[6] is used to maximise the similarity. The use of quantum mechanics-derived properties makes this alignment protocol more accurate and more efficient in describing molecular steric and electrostatic properties than conventional molecular mechanics-based methods, which use atom-centred charges and Lennard-Jones potentials. Quantum mechanics-derived properties consider important features for Computer-Aided Drug Design, such as σ-holes[7-10] and polar flattening[11-13] (responsible for halogen bonding), which cannot be described by conventional methods. Because ParaAlign is field-based, it is efficient in aligning chemically diverse ligands and finding chemically different ligands with similar binding properties, an important feature for scaffold hopping and discovery of new chemical entities to overcome patent limitations. Although ParaAlign uses quantum mechanics-derived properties, it is computationally efficient.

Parameters for Common Feature Pharmacophore (HipHop) algorithm in Discovery Studio 3.1

As the ligand conformers had been pre-computed and selected before, we set the conformer generation option to NONE. We chose hydrogen-bond acceptors, hydrogen-bond donors, hydrophobic, aromatic ring, and positive ionisable features to be used in model generation. A maximum of 25 pharmacophore models with a minimum inter-feature distance of 1.5 Å was generated during each run. Each pharmacophore model had to possess a minimum of three features and four feature points. To allow a limited number of actives to miss part of the pharmacophoric features, the number of leads that may miss, the feature misses, and the complete misses were set to “2” for D2R agonists and “1” for antagonists. For every active, the Principal and MaxOmitFeat values were set to “2”, meaning that none of the input actives is being prioritized when generating the pharmacophore model. Further, a MaxOmitFeat value of “2” for at least one compound is required when setting the complete misses value higher than zero. The other parameters were set to the default values.

Docking procedure

A representative cluster structure obtained during the simulations of the $D2^{Up}R$ model was taken for our docking procedure. To compare the binding modes of hordenine and dopamine, we removed dopamine from the binding pocket and docked hordenine, using AutoDockVina with the same parameters as above. One conformation of hordenine was selected based on the scoring function and on visual

inspection. The resulting receptor-ligand complex was submitted to energy minimisation using the SANDER module of the AMBER10 program package[14]. The all-atom force field ff99SB[15] was used for the protein residues and the general AMBER force field (GAFF)[16] for hordenine. Parameters for hordenine were assigned using antechamber[14] and the charges were calculated by means of Gaussian 09[17] at the HF/6-31(d) level and the RESP procedure according to the literature[18]. As hordenine was assumedly protonated under physiological pH, a formal charge of +1 was attributed to it. The minimisation was carried out using 2500 steps of steepest descent method followed by 7500 steps of conjugate gradient minimisation. The minimisation steps were performed in a box of TIP3P water[19] with periodic boundary conditions and a non-bonded cut-off of 10.0 Å. An appropriate number of chloride ions were added to neutralise the system.

SI - References

1. Yannai, S. *Dictionary of Food Compounds with CD-ROM*. 2nd edn, (CRC Press, 2012).
2. Clark, T. QSAR and QSPR based solely on surface properties? *J. Mol. Graph. Model.* **22**, 519-525 (2004).
3. Politzer, P. & Truhlar, D. G. *Chemical application of atomic and molecular electrostatic potentials. Reactivity, structure, scattering and energetics of organic, inorganic, and biological systems*. (Springer US, 1981).
4. Clark, T. QM Based Modelling in *Drug Design strategies: Computational techniques and applications RSC Drug discovery series No. 20* (eds T. Clark & L. Banting) 87-106 (The Royal Society of Chemistry, 2012).
5. Hodgkin, E. E. & Richards, W. G. Molecular similarity based on electrostatic potential and electric-field. *Int. J. Quantum Chem.*, 105-110 (1987).
6. Nelder, J. A. & Mead, R. A simplex method for function minimization. *Comput. J.*, 308-313 (1965).
7. Politzer, P., Lane, P., Concha, M. C., Ma, Y. G. & Murray, J. S. An overview of halogen bonding. *J. Mol. Model.* **13**, 305-311 (2007).
8. Politzer, P., Murray, J. S. & Clark, T. Halogen bonding: an electrostatically-driven highly directional noncovalent interaction. *Phys. Chem. Chem. Phys.* **12**, 7748-7757 (2010).
9. Murray, J. S., Riley, K. E., Politzer, P. & Clark, T. Directional weak intermolecular interactions: sigma-hole bonding. *Aust. J. Chem.* **63**, 1598-1607 (2010).
10. Clark, T. σ-Holes. *WIREs Comput. Mol. Sci.*, 13-20 (2013).
11. El Kerdawy, A. *et al.* Directional noncovalent interactions: repulsion and dispersion. *J. Chem. Theory Comput.* **9**, 2264-2275 (2013).
12. Stevens, E. D. Experimental electron-density distribution of molecular chlorine. *Mol. Phys.* **37**, 27-45 (1979).
13. Nyburg, S. C. Polar flattening - non-spherical effective shapes of atoms in crystals. *Acta Crystallogr. A* **35**, 641-645 (1979).
14. AMBER 10 (University of California, San Francisco, 2008).
15. Hornak, V. *et al.* Comparison of multiple amber force fields and development of improved protein backbone parameters. *Proteins: Struct., Funct., Bioinf.* **65**, 712-725 (2006).
16. Wang, J. M., Wolf, R. M., Caldwell, J. W., Kollman, P. A. & Case, D. A. Development and testing of a general amber force field. *J. Comput. Chem.* **25**, 1157-1174 (2004).
17. Gaussian 09 v. Revision B.01. (Gaussian, Inc., Wallingford, 2009).
18. Bayly, C. I., Cieplak, P., Cornell, W. D. & Kollman, P. A. A well-behaved electrostatic potential based method using charge restraints for deriving atomic charges - the resp model. *J. Phys. Chem.* **97**, 10269-10280 (1993).
19. Jorgensen, W. L., Chandrasekhar, J., Madura, J. D., Impey, R. W. & Klein, M. L. Comparison of simple potential functions for simulating liquid water. *J. Chem. Phys.* **79**, 926-935 (1983).

B – TABLE

Table S1: Molecular similarities for the tested virtual screening hits compared to the respective training set ligands expressed as Tanimoto coefficient T_c, calculated using Extended Connectivity Fingerprints 4 (ECFP4).

Compound	T_c compared to training set ligands
**D2R-agonist hits**	
Clenbuterol	0.122
Delphinidin	0.133
Fumigaclavine A	0.288
Hordenine	0.164
Kukoamine A	0.169
Leonurine	0.125
Muscimol	0.128
Pyrraline	0.094
Salsolinol	0.324
**D2R-antagonist hits**	
Ajmalicine	0.345
Dihydroberberine	0.120
Emetine	0.159
Fenpropimorph	0.110
Halofuginone	0.122
Robenidine	0.133
Roquefortine C	0.138
Sarafloxacin	0.167

C - FIGURES

Figure S1: The molecular property distributions of the newly generated screening databases depicted together with those of other types of freely available virtual screening databases.

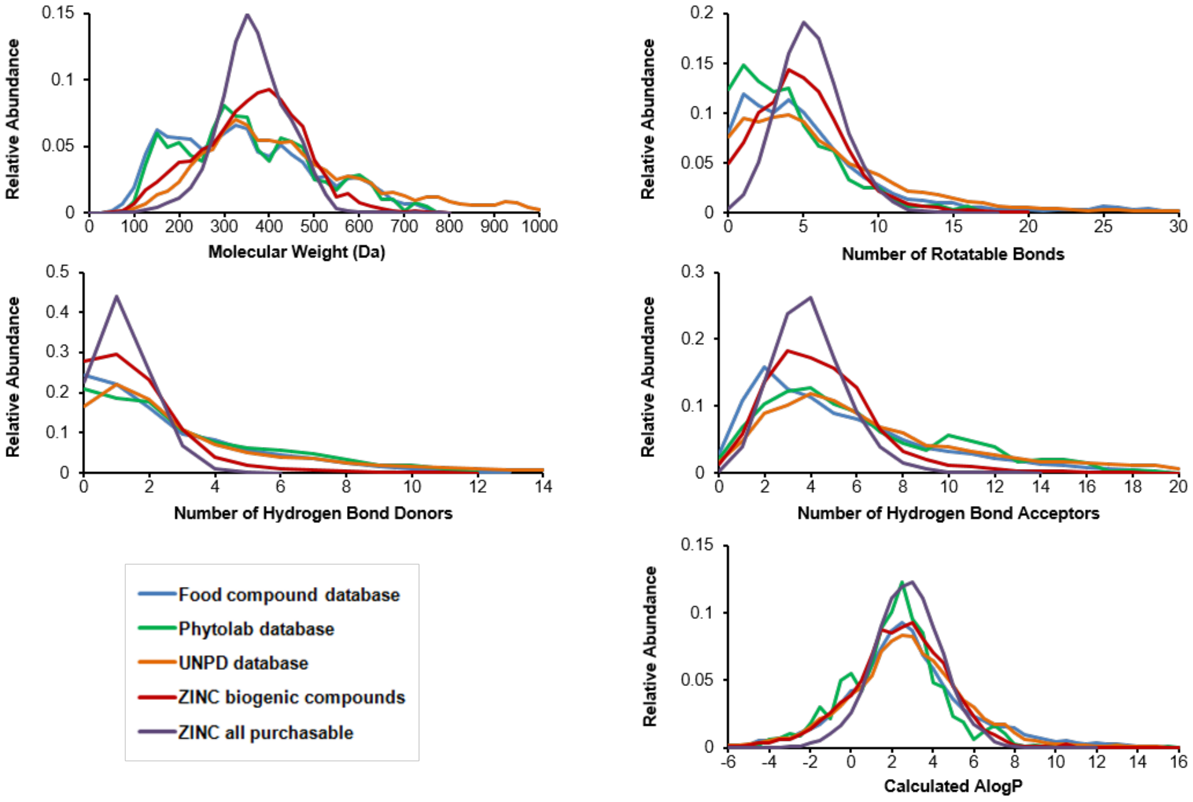

The blue lines represent the food compound database FCDB, the green lines depict PhytoLab natural products database PhyDB, the orange lines show a sample of the natural product library UNPD, the red lines a sample of the natural product library ZBC and the purple lines represent a sample of the drug-like ZAP subset. The relative abundance in the graphs indicates the number of compounds possessing a defined molecular property divided by the total number of compounds in the database or the random sample.

Figure S2: Training set ligands for D2R-agonist pharmacophore model generation.

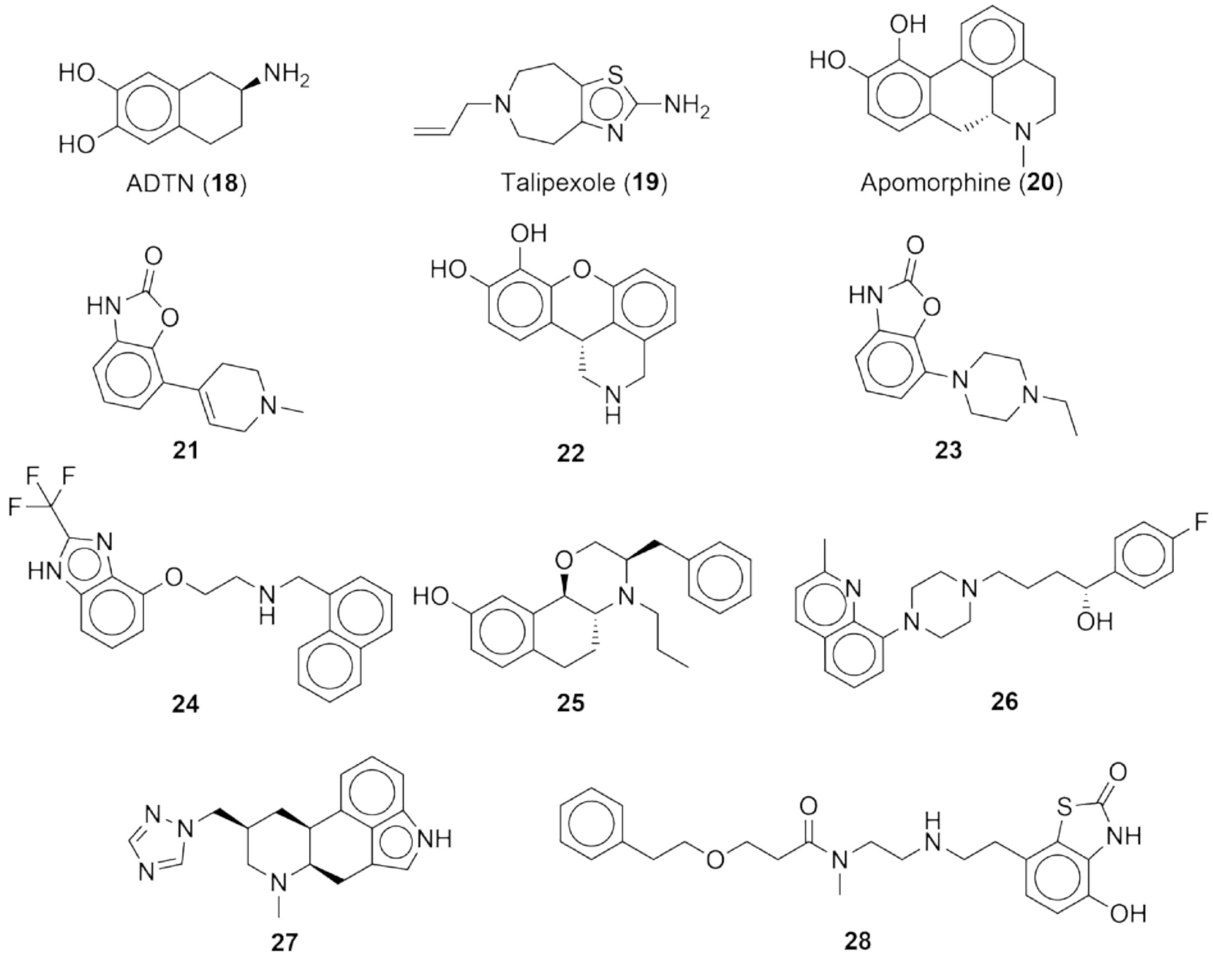

Figure S3: Training set ligands for D2R-antagonist pharmacophore model generation.

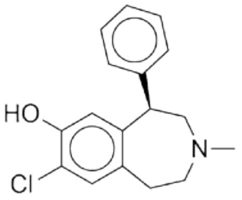

SCH23390 (**29**)

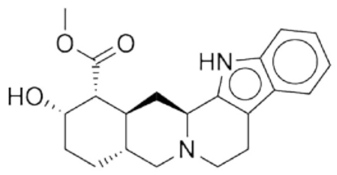

Yohimbine (**30**)

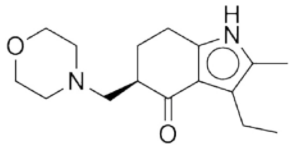

Molindone (**31**)

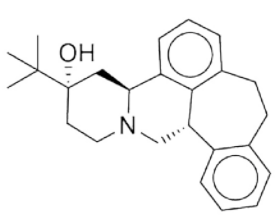

Butaclamol (**32**)

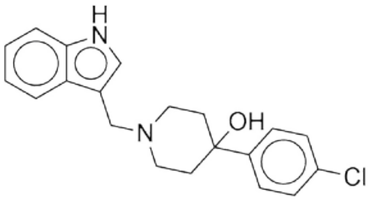

L-741,626 (**33**)

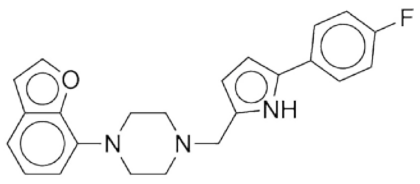

Elopiprazole (**34**)

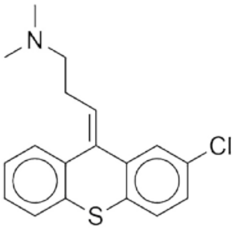

Chlorprothixene (**35**)

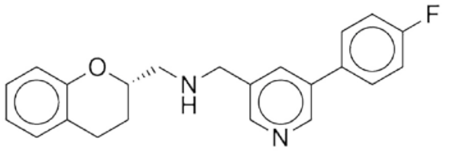

Sarizotan (**36**)

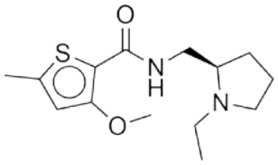

37

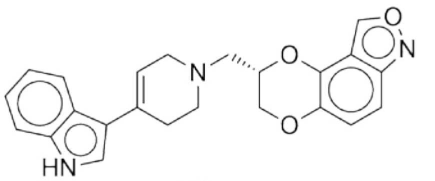

38

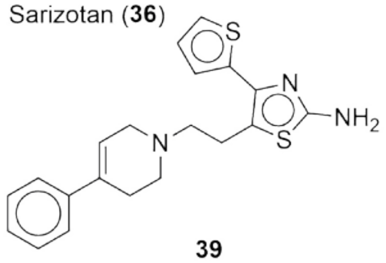

39

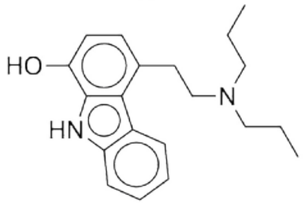

40

D – Databases

The *in silico* 3D food compound database (FCDB, containing 12,579 compounds) based on molecules selected from the Dictionary of Food Compounds (CRC Press, 2012) as well as the natural products database (PhyDB, containing 987 compounds) based on compounds from the catalogue of the vendor PhytoLab (Vestenbergsgreuth, Germany) are available online.
